# Cause-specific inequalities in mortality in Scotland: two decades of change. A population-based study

**DOI:** 10.1186/1471-2458-7-172

**Published:** 2007-07-24

**Authors:** Alastair H Leyland, Ruth Dundas, Philip McLoone, F Andrew Boddy

**Affiliations:** 1 MRC Social and Public Health Sciences Unit, 4 Lilybank Gardens, Glasgow G12 8RZ, UK; 2 Retired

## Abstract

**Background:**

Socioeconomic inequalities in mortality have increased in recent years in many countries. We examined age-, sex-, and cause-specific mortality rates for social groups in and regions of Scotland to understand the patterning of inequalities and the causes contributing to these inequalities.

**Methods:**

We used death records for 1980–82, 1991–92 and 2000–02 together with mid-year population estimates for 1981, 1991 and 2001 covering the whole of Scotland to calculate directly standardised mortality rates. Deaths and populations were coded to small areas (postcode sectors and data zones), and deprivation was assessed using area based measures (Carstairs scores and the Scottish Index of Multiple Deprivation). We measured inequalities using rate ratios and the Slope Index of Inequality (SII).

**Results:**

Substantial overall decreases in mortality rates disguised increases for men aged 15–44 and little change for women at the same ages. The pattern at these ages was mostly attributable to increases in suicides and deaths related to the use of alcohol and drugs. Under 65 a 49% fall in the mortality of men in the least deprived areas contrasted with a fall of just 2% in the most deprived. There were substantial increases in the social gradients for most causes of death. Excess male mortality in the Clydeside region was largely confined to more deprived areas, whilst for women in the region mortality was in line with the Scottish experience. Relative inequalities for men and women were greatest between the ages of 30 and 49.

**Conclusion:**

General reductions in mortality in the major causes of death (ischaemic heart disease, malignant neoplasms) are encouraging; however, such reductions were socially patterned. Relative inequalities in mortality have increased and are greatest among younger adults where deaths related to unfavourable lifestyles call for direct social policies to address poverty.

## Background

Differentials in mortality rates linked to socio-economic status have increased in several western European countries over recent decades [1]. Inequalities between geographically defined areas increased in Britain between 1979 and 1998 and were greater in Scotland than in England and Wales [2] where recent trends have provided cause for concern. Death rates for men aged 20–24 increased during the 1980s, a rise that broadened to include men aged between 20 and 34 during the 1990s; the causes contributing to these increases included suicide, drugs, alcohol and violence [3]. Their relationship to socio-economic status is unknown.

Recent (although disputed [4]) estimates of social differentials in mortality in England and Wales suggest that about half is attributable to smoking-related deaths [5]; 14% of the mortality differential in Finland is similarly attributed to alcohol-related causes [6]. The implication of these observations is that relatively straightforward social patterning provides an explanation for socio-economic differences in death rates – and, thus, the public health responses to them. The difficulty with this view is that it fails to propose an adequate explanation for the social structures and processes that underlie such outcomes of health-related behaviours.

In this paper we describe age-specific mortality rates for men and women in Scotland between 1981 and 2001 and consider the ways in which the relative importance of selected causes of death has changed. In Scotland, there are important regional differences in the distribution of social disadvantage and so the question of the extent to which unequal death rates are associated with particular localities rather than socio-economic status is also relevant. In a further analysis (for 2000–02) we describe the relative contribution of selected causes of death to inequalities at different ages.

## Methods

The data comprised death records for 1980–82, 1991–92, and 2000–02 and mid-year population estimates for 1981, 1991, 2002 provided by the General Register Office (Scotland). The information abstracted from the death records included age, sex, cause of death, and postcode sector of residence; population estimates included age, sex and area. In 2001, there were 1010 postcode sectors or part-sectors in Scotland with an average population of 5012. The number of sectors and their mean population varied slightly between time-points. The use of postcode sectors allowed a regional analysis of mortality and enabled us to divide Scotland into seven major regional groupings [7].

Each postcode sector was assigned a Carstairs Score indicative of its relative social deprivation [8-10]. These scores were derived from measures of overcrowding, male unemployment, households without a car and low social class at each of the three Censuses; they were then divided into seven deprivation categories (DepCats) for that Census. Correlation coefficients for the DepCats from the three Censuses were greater than 95%. At each Census, approximately 6% of the Scottish population lived in areas described as DepCat 1 (the least deprived) and 7% in the most deprived (DepCat 7). A change to some postcode sectors during 1990 meant that we were unable to attach deprivation scores to all deaths for this year and resulted in the restriction of this middle period to two years (1991–92) instead of three.

A second analysis employed a more recent categorisation of the socio-economic status of small areas. This was the income domain of the Scottish Index of Multiple Deprivation (SIMD) for 2004 which comprises eight measures from 2001 and 2002 relating to the receipt of social security benefits and tax credits [11]. The much smaller areas (data zones) for which this measure is provided had a mean population of 778; there were 6505 such zones. The advantage of this measure is that it permits a more detailed analysis of the contribution of particular causes of death to age-specific inequalities; however, it was not possible to analyse deaths in earlier years using this measure. The slope index of inequality (SII) was calculated for each cause and age group across quintiles of the SIMD income score. Division of the SII by national all-cause mortality for that age group then gives a relative measure of inequality [12,13].

All age standardised rates, separately for males and females, were calculated by direct standardisation to the European standard population. Causes of death were chosen to reflect changing patterns of mortality and are not comprehensive.

## Results

### Changes in mortality

The Scottish population showed a slight reduction over time, from 5,180,200 in 1981 to 5,064,200 in 2001. Table 1 details age-specific death rates from selected causes for males and females in 1980–82, 1991–92 and 2000–02. For both males and females, the death rate for children aged less than 15 reduced by about 30% in each inter-censal period and by 2000–02 were less than half the rates in 1980–82. At older ages, the major reduction was between ages 45 and 59 where death rates in men were 37% less and 34% less in women in 2000–02 when compared to 1980–82. Death rates for men aged 60–74 declined by 34% and those for women by 28% over these two decades. In the younger age groups, the reduction was greater between 1980–82 and 1991–92; at ages 60–74, the greater fall was between 1991–92 and 2000–02.

**Table 1:** Age specific mortality for selected causes. Rates per 100,000 men and women, Scotland, 1980–82, 1991–92 and 2000–02.

		**males**			**females**		
			
	**Age**	**1980–82**	**1991–92**	**2000–02**	**1980–82**	**1991–92**	**2000–02**
**All causes**	**0–14**	135	87	60	102	62	46
	**15–29**	97	101	111	38	38	38
	**30–44**	208	176	202	129	104	101
	**45–59**	1,072	794	672	617	483	407
	**60–74**	4,138	3,464	2,737	2,282	2,034	1,639
	**75+**	14,786	12,675	10,863	10,025	8,792	8,214
		
	**all ages**	1,396	1,159	976	856	736	646

**Ischaemic Heart Disease **(ICD9 410–414; ICD10 I20-25)	**0–14**	0	0	0	0	0	0
	**15–29**	1	1	1	0	0	0
	**30–44**	45	28	20	9	7	5
	**45–59**	433	281	156	114	82	40
	**60–74**	1,504	1,219	695	691	554	305
	**75+**	3,926	3,417	2,519	2,432	2,233	1,671
		
	**all ages**	434	345	220	205	174	113

**All malignant neoplasms **(ICD9 140–208; C00-97)	**0–14**	6	3	3	4	4	2
	**15–29**	9	6	6	8	6	5
	**30–44**	37	32	25	51	44	33
	**45–59**	281	248	203	254	235	191
	**60–74**	1,145	1,127	990	651	712	644
	**75+**	2,523	2,651	2,568	1,274	1,366	1,505
		
	**all ages**	305	300	269	193	198	184

**Chronic liver disease **(ICD9 571; ICD10 K70, K73-74)	**0–14**	0	0	0	0	0	0
	**15–29**	0	0	1	0	0	0
	**30–44**	6	8	19	5	5	8
	**45–59**	23	23	62	13	15	30
	**60–74**	31	32	74	18	20	29
	**75+**	19	17	31	13	11	18
		
	**all ages**	10	11	27	6	7	12

**Intentional self harm & events of undetermined intent **(ICD9 E950-959, 980–989; ICD10 X60-84, Y87.0, Y10-Y34, Y87.2)	**0–14**	0	0	1	0	8	1
	**15–29**	17	29	37	5	9	9
	**30–44**	26	31	41	12	11	12
	**45–59**	30	25	28	21	9	10
	**60–74**	26	21	23	15	10	9
	**75+**	25	24	20	12	8	7
		
	**all ages**	19	21	26	10	7	8

**Mental and behavioural disorders due to use of drugs **(ICD9 304, 305.2-.9; ICD10 F11-16, F18-19)	**0–14**	0	0	0	0	0	0
	**15–29**	1	3	22	0	1	5
	**30–44**	0	1	18	0	0	3
	**45–59**	0	0	3	0	0	0
	**60–74**	0	0	0	0	0	0
	**75+**	0	0	0	0	0	0
		
	**all ages**	0	1	9	0	0	2

**Mental and behavioural disorders due to use of alcohol **(ICD9 291, 303, 305.0; ICD10 F10)	**0–14**	0	0	0	0	0	0
	**15–29**	1	0	1	0	0	0
	**30–44**	5	5	7	2	2	3
	**45–59**	11	11	23	5	4	8
	**60–74**	11	8	22	5	3	7
	**75+**	4	4	8	1	3	2
		
	**all ages**	5	5	9	2	2	3

A more complex pattern is evident for deaths aged between 15 and 44 years. At ages 15–29, the male death rate increased by 4% in the first decade and by a further 10% in the second whilst the female death rate remained approximately constant. For males aged between 30 and 44, the rate declined by 16% in the first decade but then increased by a similar proportion between 1991–92 and 2000–02 so that the rate at the end of the period was little changed from that of 1980–82. For women, the reduction in the earlier decade was 19% but only 3% in the second.

These substantial reductions in all-cause death rates reflect changes in certain major causes. Deaths attributed to ischaemic heart disease (IHD) were 49% fewer for men and 45% fewer for women between 1980–82 and 2000–02; these reductions were particularly marked at ages 45–59 where the reduction was 64% for either sex. Reductions in deaths from all malignant neoplasms were lower (12% for men and 5% for women) but were also somewhat greater under the age of 60. The death rates for the remaining causes are considerably lower than those for the major causes of death described above, but these causes show substantial increases, especially in the decade between 1991–92 and 2000–02. With the exception of alcohol-related deaths, this pattern of increasing rates was much less evident for women.

### Regional differences

Regional differences in all cause and cause specific mortality for males and females aged less than 65 are described in Table 2. In 2000–02, most Scottish regions had rates that were close to the national rate although the North East had consistently lower rates. The other exception was Clydeside where the reduction in mortality rates was less than that for other regions with the consequence that, by 2000–02, mortality in this region was 30% above the Scottish average having been only 17% higher in 1980–82. This divergence between Clydeside and the rest of Scotland is explained by changing death rates for IHD, chronic liver disease in males, and behavioural disorders due to the use of drugs.

**Table 2:** Age standardised mortality for selected causes and for major regions of Scotland. Rates per 100,000 men and women aged 0–64, 1980–82, 1991–92 and 2000–02.

		**males**			**females**		
			
	**Region**	**1980–82**	**1991–92**	**2000–02**	**1980–82**	**1991–92**	**2000–02**
**All causes**	**H&I**	478	359	308	257	202	178
	**NE**	401	318	280	235	199	157
	**Clyde**	577	474	439	326	269	229
	**Central**	476	374	306	292	222	185
	**East**	431	346	305	261	220	175
	**SW**	498	362	311	288	229	193
	**SE**	427	354	298	250	210	177

**Ischaemic Heart Disease **(ICD9 410–414; ICD10 I20-25)	**H&I**	167	388	55	35	232	19
	**NE**	129	102	45	36	27	12
	**Clyde**	192	86	82	60	30	26
	**Central**	158	141	63	54	48	16
	**East**	147	117	60	48	39	19
	**SW**	182	101	65	54	34	20
	**SE**	137	117	55	41	42	13

**All malignant neoplasms **(ICD9 140–208; C00-97)	**H&I**	102	95	91	100	30	75
	**NE**	98	115	83	91	39	71
	**Clyde**	137	92	108	111	92	85
	**Central**	110	89	85	97	83	75
	**East**	109	130	88	96	107	74
	**SW**	115	102	81	101	89	74
	**SE**	106	96	87	89	92	78

**Chronic liver disease **(ICD9 571; ICD10 K70, K73-74)	**H&I**	7	95	13	3	93	7
	**NE**	5	105	11	3	92	4
	**Clyde**	13	107	42	8	95	15
	**Central**	6	8	21	5	5	11
	**East**	4	6	17	3	3	7
	**SW**	7	16	17	4	9	11
	**SE**	8	8	17	5	5	11

**Intentional self harm & events of undetermined intent **(ICD9 E950-959, 980–989; ICD10 X60-84, Y87.0, Y10-Y34, Y87.2)	**H&I**	23	3	30	9	4	7
	**NE**	18	8	22	10	6	6
	**Clyde**	22	6	29	11	3	9
	**Central**	14	9	24	8	6	6
	**East**	16	26	26	9	6	8
	**SW**	16	18	27	8	5	7
	**SE**	16	25	22	9	7	10

**Mental and behavioural disorders due to use of drugs **(ICD9 304, 305.2-.9; ICD10 F11-16, F18-19)	**H&I**	0	18	4	0	5	0
	**NE**	0	20	11	0	8	2
	**Clyde**	0	17	17	0	6	3
	**Central**	0	21	4	0	7	1
	**East**	0	21	6	0	7	1
	**SW**	0	0	9	0	0	2
	**SE**	0	0	7	0	0	1

**Mental and behavioural disorders due to use of alcohol **(ICD9 291, 303, 305.0; ICD10 F10)	**H&I**	4	1	12	1	0	6
	**NE**	3	2	10	1	0	4
	**Clyde**	8	2	12	3	1	3
	**Central**	1	0	4	0	0	2
	**East**	3	1	8	2	0	3
	**SW**	3	1	6	1	0	2
	**SE**	3	6	7	2	2	3

Key: H&I: Highlands and Islands; NE: North East; Clyde: Clydeside conurbation; SW: South West; SE: South East

### Deprivation

Table 3 sets out the same age-standardised rates for males and females at ages 0–64 but divided between the seven DepCats based on Carstairs scores from each Census [8-10]. The national reduction in all-cause mortality of 32% was exceeded in the more affluent categories (39–49% for DepCats 1–3) with more modest reductions in the more deprived localities. This general pattern contrasts with the rate for the most deprived DepCat 7 where death rates for men aged less than 65 increased by 11% between 1991–92 and 2000–02. The ratio of deaths in DepCat 1 to those in DepCat 7 in 1981 was 1:2.3; by 1991 the ratio had increased to 1:2.8, and to 1:4.4 in 2001. Death rates in 2001 for men aged less than 65 and living in DepCats 4–7 (that is, about 60% of the male population) were higher than those living in DepCat 1 20 years earlier. Put another way, despite a twenty-year reduction of 31% in overall death rates, the mortality rate for men under the age of 65 and living in DepCat 7 localities in 2001 was 44% greater than the Scottish rate for 1981.

**Table 3:** Age standardised mortality for selected causes and for area deprivation category in Scotland. Rates per 100,000 men and women aged 0–64, 1980–82, 1991–92 and 2000–02.

		**males**			**females**		
			
	**DEPCAT**	**1980–82**	**1991–92**	**2000–02**	**1980–82**	**1991–92**	**2000–02**
**All causes**	**1**	316	226	161	185	144	124
	**2**	383	273	210	225	184	134
	**3**	443	331	272	261	188	159
	**4**	484	379	329	282	225	192
	**5**	543	440	413	314	255	224
	**6**	618	506	479	358	288	253
	**7**	719	634	705	393	380	344

**Ischaemic Heart Disease **(ICD9 410–414; ICD10 I20-25)	**1**	115	68	26	28	16	8
	**2**	127	73	38	34	20	9
	**3**	156	97	52	46	27	14
	**4**	166	117	66	48	38	18
	**5**	186	134	81	60	46	26
	**6**	209	151	88	69	56	29
	**7**	202	181	128	72	72	45

**All malignant neoplasms **(ICD9 140–208; C00-97)	**1**	82	66	59	80	75	61
	**2**	96	83	68	87	89	66
	**3**	101	94	82	96	86	72
	**4**	116	102	89	103	93	79
	**5**	129	117	106	103	104	81
	**6**	143	142	119	116	104	91
	**7**	170	157	151	114	124	108

**Chronic liver disease **(ICD9 571; ICD10 K70, K73-74)	**1**	4	3	5	3	2	4
	**2**	6	5	8	4	2	4
	**3**	7	7	13	4	4	7
	**4**	8	8	19	5	5	11
	**5**	8	10	33	7	9	15
	**6**	14	15	42	10	10	16
	**7**	19	16	80	9	12	27

**Intentional self harm & events of undetermined intent **(ICD9 E950-959, 980–989; ICD10 X60-84, Y87.0, Y10-Y34, Y87.2)	**1**	8	10	11	6	4	4
	**2**	15	18	17	9	5	5
	**3**	16	19	21	8	5	6
	**4**	18	18	26	10	7	8
	**5**	17	21	32	10	8	10
	**6**	24	27	37	10	7	11
	**7**	35	44	45	17	17	16

**Mental and behavioural disorders due to use of drugs **(ICD9 304, 305.2-.9; ICD10 F11-16, F18-19)	**1**	0	0	2	0	0	1
	**2**	0	0	4	0	0	0
	**3**	0	0	4	0	0	1
	**4**	0	0	8	0	0	1
	**5**	0	1	10	0	0	1
	**6**	0	1	19	0	0	4
	**7**	1	2	40	0	2	8

**Mental and behavioural disorders due to use of alcohol **(ICD9 291, 303, 305.0; ICD10 F10)	**1**	1	1	1	3	1	2
	**2**	2	1	3	1	1	2
	**3**	3	4	6	1	2	2
	**4**	3	5	8	1	2	3
	**5**	4	7	13	2	3	3
	**6**	9	8	14	3	1	4
	**7**	16	4	22	7	2	8

Similar patterns of change for female deaths meant that the ratio of DepCat 1 to DepCat 7 mortality rates was 1:2.1 in 1981 and 1:2.8 in 2001. Similar observations apply to specific causes of death; over the two decades, a reduction of 70% in IHD deaths compares to only 37% for DepCat 7. Although the social gradient is less pronounced for deaths from malignant neoplasms, the reduction in DepCat 1 was 29% and that for DepCat 7 only 11%.

Rather more complex patterns are evident for the other causes of death set out in Table 3. Deaths from male suicides, although four times more common in DepCat 7 than in DepCat 1, show approximately equal increases in the different deprivation categories. On the other hand, for both males and females, there were increasingly strong social gradients for drug and alcohol related deaths. For DepCat 7, the 2001 rate for chronic liver disease was four times greater than that for 1981 and 16 times greater than the rate for DepCat 1. Although the actual rates are much lower, the trend for women is very similar.

### Deprivation and region

The Clydeside region has an excess of deprivation compared to the rest of Scotland. Table 4 shows that 40% of the region's population lived in the most deprived two groups (DepCats 6 and 7) in 2001 compared to 18% nationally and just 6% in the North East. The combination of excess mortality in the Clydeside region and the higher rates experienced by more deprived localities raises the question of the extent to which the former is explained by the latter. Table 5 sets out death rates for the seven DepCats in each region: against an overall excess mortality of 30% in 2001, the rates for Clydeside are in line with the Scottish average in the least deprived areas and have an excess mortality of the order of 7–8% in DepCats 4, 5 and 6. No localities in the North East were included in DepCat 7 but for the remainder – with the exception of DepCat 3 – the male mortality rate in this region in 2001 was higher than that of the Clydeside conurbation. For women, within each DepCat the mortality rate in the Clydeside region was within 6% of the Scottish rate. Excess mortality in Clydeside can mostly be attributed to the poorer experience of socially disadvantaged populations.

**Table 4:** The distribution of the 2001 Scottish population across major regions of Scotland, and the percentage of the area's population in each deprivation category.

		**Deprivation category (DepCat)**						
	
**Region**	**Population**	1	2	3	4	5	6	7
Highlands and Islands	367,850	1	9	45	36	8	1	0
North East	525,850	18	26	26	22	4	6	0
Clydeside conurbation	1,447,870	5	10	10	16	19	20	20
Central	438,570	5	18	12	46	16	3	0
East	738,550	6	17	27	21	14	11	4
South West	818,410	1	8	27	33	16	15	0
South East	727,100	9	16	26	25	16	4	3

**All Scotland**	5,064,200	6	14	22	25	15	11	7

**Table 5:** All cause mortality by deprivation category for major regions of Scotland. Rates per 100,000 men and women aged 0–64, 1980–82, 1991–92 and 2000–02.

		**males**			**females**		
			
**DEPCAT**	**Region**	**1980–82**	**1990–92**	**2000–02**	**1980–82**	**1990–92**	**2000–02**
	**H&I**	342	315	244	114	172	132
	**NE**	290	217	170	170	177	135
	**Clyde**	330	221	161	201	135	119
**1**	**Central**	304	309	125	150	111	90
	**East**	318	238	199	162	146	140
	**SW**	349	198	140	210	76	111
	**SE**	296	225	132	187	145	119

	**H&I**	425	276	221	249	171	145
	**NE**	367	247	218	217	160	132
	**Clyde**	386	287	215	229	204	140
**2**	**Central**	377	288	202	226	220	138
	**East**	366	289	204	202	195	135
	**SW**	420	257	240	221	194	139
	**SE**	365	282	190	239	155	116

	**H&I**	445	334	290	265	190	169
	**NE**	406	299	272	254	171	154
	**Clyde**	454	374	281	277	196	168
**3**	**Central**	439	436	265	295	196	168
	**East**	422	326	259	257	197	150
	**SW**	479	327	254	265	197	155
	**SE**	426	288	289	232	171	161

	**H&I**	564	403	334	281	199	200
	**NE**	446	370	371	248	223	179
	**Clyde**	534	449	352	297	222	196
**4**	**Central**	491	329	341	321	215	202
	**East**	462	361	304	272	232	176
	**SW**	472	344	302	297	251	190
	**SE**	447	407	329	267	222	197

	**H&I**	661	521	390	192	312	186
	**NE**	592	416	497	324	262	242
	**Clyde**	565	446	445	315	252	230
**5**	**Central**	511	416	412	298	228	226
	**East**	489	430	386	338	238	206
	**SW**	554	428	375	323	272	234
	**SE**	513	462	410	287	258	224

	**H&I**	913	452	846	350	100	88
	**NE**	392	435	583	277	249	260
	**Clyde**	646	518	515	368	293	255
**6**	**Central**	628	782	388	318	338	261
	**East**	506	466	445	369	291	249
	**SW**	591	488	425	351	276	262
	**SE**	561	411	403	300	302	220

	**H&I**	607	0		0	0	
	**NE**						
	**Clyde**	735	635	717	403	382	340
**7**	**Central**						
	**East**	557	2547	623	303	0	307
	**SW**	794	544		206	268	
	**SE**	697	666	675	380	397	455

Key: H&I: Highlands and Islands; NE: North East; Clyde: Clydeside conurbation; SW: South West; SE: South East

### Changes in inequalities

Table 6 compares ratios of the mortality rates in the most deprived areas (DepCats 6 and 7) and the most affluent areas (DepCats 1 and 2) with the intermediate areas (DepCats 3–5) comprising 62% of the Scottish population. Thus in 1980–82 the all cause mortality rate among men aged under 65 living in the most affluent areas was 25% below the rate for DepCats 3–5 while the mortality rate in the most deprived areas was 35% higher. By 2000–02 the equivalent rates were 41% lower and 71% higher respectively, suggesting a widening relative differential. Such a pattern was seen for most of the causes reported for men; for women the widening inequalities were largely restricted to IHD and suicide.

**Table 6:** Mortality rate ratios for deprivation category groups relative to categories 3–5. Men and women aged 0–64, Scotland 1980–82, 1991–92 and 2000–02.

		**Rate ratio relative to deprivation categories 3–5**					
			
		**Men**			**Women**		
			
	**Deprivation categories**	**1980–82**	**1991–92**	**2000–02**	**1980–82**	**1991–92**	**2000–02**
**All causes**	1–2	0.75	0.69	0.59	0.75	0.78	0.70
	6–7	1.35	1.47	1.71	1.31	1.47	1.53
**Ischaemic heart disease**	1–2	0.74	0.63	0.53	0.64	0.51	0.48
	6–7	1.23	1.42	1.59	1.38	1.70	1.88
**All malignant neoplasms**	1–2	0.80	0.75	0.72	0.84	0.91	0.83
	6–7	1.34	1.43	1.44	1.14	1.20	1.26
**Chronic liver disease**	1–2	0.69	0.54	0.34	0.75	0.33	0.41
	6–7	2.11	1.80	2.75	1.98	1.86	1.95
**Intentional self harm & events of undetermined intent**	1–2	0.76	0.82	0.59	0.87	0.73	0.63
	6–7	1.64	1.76	1.56	1.32	1.63	1.69
**Mental and behavioural disorders due to use of drugs**	1–2	1.26	0.50	0.43	0.00	0.00	0.42
	6–7	2.79	2.35	3.82	5.89	6.22	4.76
**Mental and behavioural disorders due to use of alcohol**	1–2	0.47	0.27	0.30	1.28	0.58	0.68
	6–7	3.44	1.31	1.98	2.81	0.73	1.71

### Cause-specific impact on inequalities

**Figure 1:**
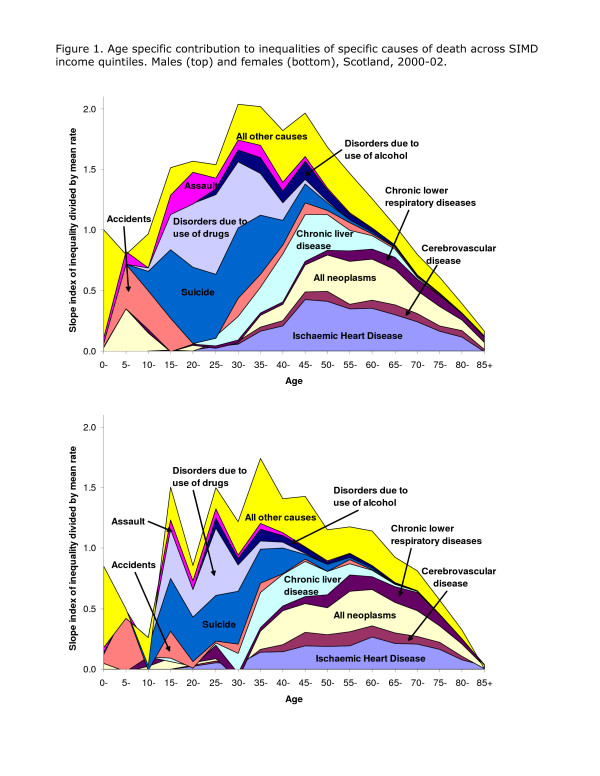


The conclusions of the analysis of death rates linked to the income domain of SIMD are summarised in Figure 1 which comprises stacked graphs of the SII divided by the appropriate Scottish mortality rate for males and females in 2000–02, developing methods used elsewhere [14]. The total enclosed area indicates the relative difference in mortality rates between the least and most deprived areas: a value of zero indicates that there is no inequality, a value of one suggests that the difference between the least and most deprived areas is equal to the mean mortality rate. In other words, a value of one means that the rate in the most deprived areas is about 50% greater than the average and about 50% lower in the least deprived areas. The maximum value for this measure is about two; for males, the measure takes a value close to one at ages 0–14, increases to about 1.5 between ages 15–29 and increases to 2 between ages 30–49. There is then a steady decrease in inequalities with increasing age but even at age 80–84 the inequality measure is 0.4 and 0.2 at greater ages.

The width of the different bands indicates the contribution of different causes to overall inequality. Suicide is a significant cause of unequal mortality from ages 10–14 and makes an important contribution up to about age 40 when chronic liver disease, IHD and neoplasms become the major explanations. Disorders due the use of drugs make a substantial contribution in both sexes between the ages of 15 and 50, with assault also contributing to the excess mortality of young males. Although the pattern of inequalities for women is essentially the same as that for men, their relative inequality is somewhat less in each age group; chronic liver disease becomes important at rather later ages and the significance of drug-related deaths is also less. Cerebrovascular disease and chronic lower respiratory disease are more important for relative inequalities in women than in men which – for these causes – are greater at younger ages.

## Discussion

To a large extent, the general decline in Scottish death rates over the past two decades can be attributed to reductions in what are usually regarded as the major causes of death – chiefly, ischaemic heart disease, cerebrovascular disease and, to a lesser degree, malignant neoplasms. These reductions in mortality have been greatest in socially advantaged groups in the population but are also apparent in more deprived localities and so the question of the extent to which persisting inequalities simply represent a lag for socially disadvantaged groups within a more general process of decline becomes pertinent. The argument that this is not so is supported by two principal considerations: the first, and more disturbing, is the observation that inequalities are increasing for many causes and are greatest at younger ages with the substitution of "newer" causes (suicide, drugs, alcohol and assault) in the most deprived neighbourhoods. The second is that the decline in death rates in different Carstairs categories is inconsistent: as an illustration, the IHD death rate in DepCat 1 reduced by 73% between 1980–82 and 2000–02 and that for DepCat 2 by 70%. The corresponding reduction for DepCat 7 was 36%. Additional support for the view that these differences are underpinned by unequal social circumstances is found in the extent to which the different rates seen in each DepCat can explain differences in death rates between different parts of Scotland. It is clear from Table 4 that for the most part mortality rates in the Clydeside conurbation are not so different from those for the general population within each deprivation band. This means that the considerable excess mortality of the Clydeside region – mortality in the region was 29% above the Scottish rate in 2000–02 [7] – derives from the greater prevalence of social disadvantage in Clydeside. The proportion of the population of the region that lives in relative deprivation has changed little over time, and so the fact that the excess mortality in Clydeside has increased from 17% in 1980–82 and 22% in 1991–92 is likely to be due to the increasing differences in mortality rates between the deprivation groups.

Our findings concur with the increasing socioeconomic inequalities seen in other countries up to the end of the 1990s based on individual measures of education [1,15,16] or socioeconomic status derived from occupation [1,17]. Such increases were for the most part attributable to changes in the rates of cardiovascular disease, with declines being steepest among the most privileged groups. There are particularly pertinent comparisons to be drawn between the results we present for Scotland and the detailed published analysis of mortality rates in Estonia, where declining all cause mortality rates among men aged 20–39 with University education were offset by increases among men with lesser education resulting in an overall increase of 25% in this age group between 1989 and 2000, and where differential increases in alcohol-related mortality contributed significantly to the widening inequalities [15].

Results based on individual socioeconomic status (not presented here) were inconclusive for three principal reasons. Firstly, there has been a change in the coding of social class between 1991 and 2001 from the Registrar General's Social Class to the National Statistics Socioeconomic Classification (NS-SEC) making comparisons over time difficult. Secondly, a large proportion of deaths and of the population at certain ages cannot be allocated to a social class and it is difficult to understand the "not classified" category. Finally, there was a considerable mismatch between the proportion of the population not classified on death records and in the general population, with the problem being more pronounced at younger and older ages and for women [7].

The area deprivation measures employed indicate relative deprivation, identifying proportions of the population living in the most deprived areas at each time. This raises the question as to whether the finding of increasing inequalities between the different deprivation categories truly reflects increasing inequalities between equivalent groups or whether selective population migration has led to an increased polarisation of Scottish society, with the deprived population becoming more deprived over time. Although it is impossible to answer this with certainty, we can find clues about the population change by considering the characteristics that comprise the Carstairs index [9,10]. Between 1991 and 2001 male unemployment fell from 35% to 19% in DepCat 7 compared to a decline from 13% to 8% in Scotland as a whole. This implies a slightly greater relative decline in unemployment in DepCat 7 than was seen in the rest of the country. However, the proportion of low social class fell from 21% to 18% throughout Scotland whilst the decline in DepCat 7 was just from 33% to 32%. With the declines in overcrowding and households without a car being approximately equivalent in DepCat 7 and the rest of Scotland an increase in deprivation in DepCat 7 is not obvious.

In 2000 the system of coding of deaths in Scotland changed from the 9^th ^to the 10^th ^revision of the International Classification of Diseases (ICD-9 to ICD-10). Such a change may influence the way in which particular deaths are coded with implications for trends in specific causes of death. The trends may also be influenced by incidental changes in coding rules applied in Scotland and in changes in the reporting of causes of death on death certificates. Although it is difficult to quantify the exact impact of such changes, recent European comparisons suggested that whilst a discontinuity associated with a change in ICD coding was discernible in about 10% of cases, the impact of the change in classification from ICD-9 to ICD-10 was small compared to the magnitude of the changes in death rates and in inequalities associated with particular causes shown in this paper [18]. Similarly, in the United States changes in ICD classification system were found to have minimal impact on trends [19]. However, these reports were restricted to broad groups of causes of death or to the causes most common among older age groups. So what of the other causes considered in this paper? It has been suggested that the rise in deaths due to liver cirrhosis were evident over a long period of time and could not be artefacts of the coding change [20]. Similarly, trends in deaths due to suicide in the United Kingdom [21] and drug- and alcohol-related deaths in England and Wales [22,23] did not show marked differences following the coding changes with the exception of mental and behavioural disorders due to the use of alcohol which showed a decline of 11% among women. A bridge-coding analysis in Scotland identified few changes following the introduction of ICD-10 with the exception of a decrease in the number of deaths assigned to the alcohol abuse code [24]. The lack of change associated with the new coding system does not mean that there have not been changes in coding rules that have led to more gradual shifts in the coding of deaths. If the coding of particular causes has changed over time then it is possible that the changes may differentially affect one population group more than another leading to apparent changes in inequalities. However, such coding changes will not, of course, influence the figures for all cause mortality. So widening inequalities in all cause mortality must reflect increasing inequalities in certain causes; any uncertainty is over the extent of the increase for individual causes.

## Conclusion

Without denying the importance of specific public health interventions – such as those directed at smoking and alcohol consumption – it is difficult to escape the conclusion that the inequalities we describe have more fundamental origins in lifestyles determined by poverty. The improvement in IHD mortality over 20 years in the more affluent areas implies an ability to adopt healthier life-styles and behaviours. The reverse of this coin, characterised by a growing number of young deaths from essentially negative lifestyles, is evidence of a need for social policies which have a more direct influence on poverty and its correlates.

The problems we describe and the question as to how to formulate policy to address health inequalities are not new and are not limited to Scotland. There is little evidence available regarding the effectiveness of policies [25] or interventions [26] to inform the creation of such policies. The lack of evidence does not mean that the issue has not been addressed; the Independent Inquiry into Inequalities in Health in the UK was sufficiently persuaded by the likelihood of a link between low income and poor health to say that 'without a shift of resources to the less well off ... little will be accomplished in terms of a reduction in health inequalities by interventions addressing particular "downstream" influences' and to recommend policies to reduce income inequalities [27]. The WHO Commission on Social Determinants of Health will recommend policies designed to improve the health of the world's most vulnerable people; they take the view that 'if the major determinants of health are social, so must be the remedies' [28]. It is encouraging that the Scottish Executive has recognised these issues [29] but the scope of the devolution settlement is limited in terms of its ability to tackle the fundamentals underlying deprivation. Tackling health inequalities and their causes is not solely the preserve of health policy [28] and there is a case for more effective action from central governments.

## Competing interests

The author(s) declare that they have no competing interests.

## Authors' contributions

AL participated in the design and analysis of this study and took primary responsibility for the writing of the paper. All of the other authors contributed to the writing of the report. PMcL and AB participated in the design of the study. RD and PMcL participated in the data gathering and analysis.

## Pre-publication history

The pre-publication history for this paper can be accessed here:


